# Extrauterine leiomyomas in uncommon locations: two case reports and literature review

**DOI:** 10.3389/fmed.2024.1408247

**Published:** 2024-07-16

**Authors:** Onur Yavuz, Ali Hakan Kula, Zeynep Bayramoğlu, Nur Yağ mur Aydin, Kadir Alper Mankan, Aslı Akdöner

**Affiliations:** ^1^Department of Obstetrics and Gynecology, Dokuz Eylül University School of Medicine, Izmir, Türkiye; ^2^Department of Pathology, Dokuz Eylül University School of Medicine, Izmir, Türkiye

**Keywords:** Bartholin's cyst, cystocele, leiomyosarcoma, vaginal cysts, vaginal leiomyoma, vulvar leiomyoma

## Abstract

**Objectives:**

This study aims to provide an overview of the diagnosis, treatment, and follow-up management of vulvar and vaginal leiomyomas through the presentation of two rare cases.

**Methods:**

Detailed clinical presentations, surgical procedures, histopathological examinations, and follow-up outcomes of two cases of vulvar and vaginal leiomyomas are described. Relevant literature is also reviewed to contextualize the findings.

**Results:**

Both patients underwent successful surgical excision of the leiomyomas with no perioperative or postoperative complications. Histopathological examinations confirmed the diagnosis of leiomyoma based on characteristic microscopic features and immunohistochemical analyses.

**Conclusion:**

Vulvar and vaginal leiomyomas are rare benign tumors that require careful evaluation for accurate diagnosis and appropriate management. Surgical excision remains the primary treatment modality, and long-term follow-up is essential for monitoring recurrence and ensuring favorable outcomes.

## 1 Introduction

In the vulvar region, a wide range of benign, premalignant, and malignant tumors can arise. In reproductive-age women, unilateral swelling of the vulva with a mass is commonly regarded as a Bartholin's cyst (1, 2). While uterine leiomyomas are quite common, vulvar leiomyomas, frequently misinterpreted as Bartholin's cysts, are very rare (3). They constitute approximately 0.03% of all gynecological neoplasms and 0.07% of all vulvar tumors (4). Although case reports or series have been reported in the literature, there is no specific guideline determining the management approach of vulvar leiomyoma.

Hemangioma, papilloma, mucosal polyp, and leiomyoma are among the types of vaginal tumors that are seldom encountered (5). Vaginal leiomyomas are benign mesenchymal tumors with a very low incidence. Diagnosis is generally confirmed postoperatively through histological examination of the mass. Such tumors usually arise from the anteriorivaginal wall and are therefore often misdiagnosed as cystocele (5). They can cause various clinical presentations, even leading to damage to adjacent organs (5, 6).

In this article our aim was to provide an overview of the diagnosis, treatment, and follow-up management of vulvar and vaginal leiomyomas through the presentation of two rare cases.

## 2 Case presentations

### 2.1 Case 1. Vulvar leiomyoma masquerading as a Bartholin's cyst

**Figure 1:**
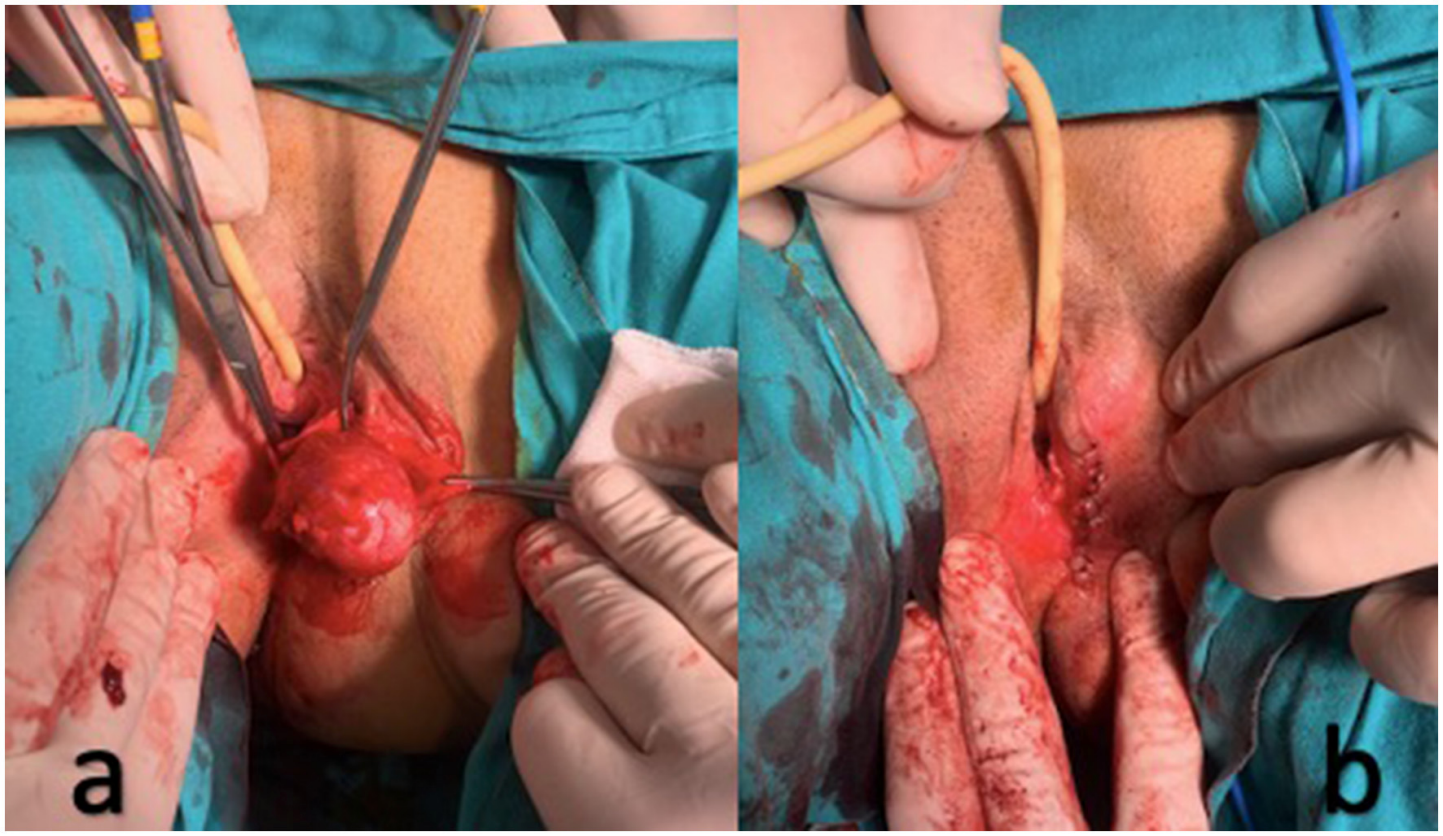
**(a)** A soft, fleshy, well-defined mass (leiomyoma); **(b)** The mass (leiomyoma) was excised intact.

A 42-year-old female patient, gravida 2, parity 2, presented to the gynecology clinic with a history of a mass on the left labial area and complaints of dyspareunia persisting for 1 year. There was no history of discharge, fever, weight loss, or a history of malignancy in the family. She had previously undergone two cesarean sections. The general examination revealed no abnormalities except for a soft mass measuring 3.5 × 3 × 2 cm in the left labial area, located medially to the left labia minora. At first, the tumor was identified as a Bartholin'sicyst. A soft, meaty, well-defined lump was visible via the mucocutaneous junction incision made while under spinal anesthesia in the lithotomy position. Theimass was excised intact and taken for histopathological analysis (Figures 1a, b). Total operation time was 30 min. No perioperative and postoperative complications were observed. The patient was discharged on the first postoperative day. She made a complete recovery, returning to her regular activities the next day. The symptoms at the time of admission have completely resolved. In the 6-month follow-up period, no recurrence was observed.

### 2.2 Histopathological examination for Case 1

**Figure 2:**
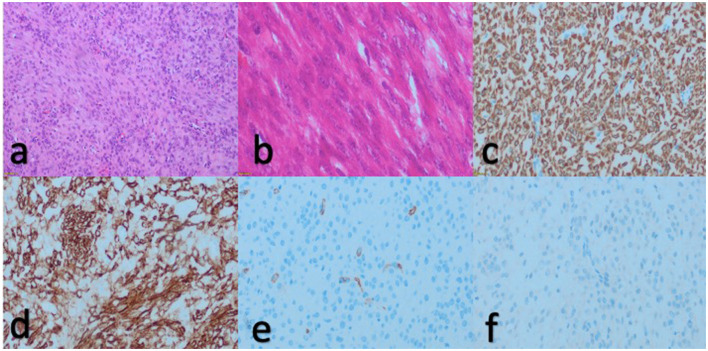
Hematoxylin and eosin staining revealed intersecting fascicles of monotonous spindle cells with normocellular, eosinophilic cytoplasm, cigar-shaped nuclei, and diminutive nucleoli under microscopic examination [**(a)**, 20x and **(b)**, 40x]. Immunohistochemical examination showed positive reaction with desmin [**(c)**, 20x] and h-caldesmom [**(d)**, 20x], while S100 **[(e)**, 20x] and CD34 [**(f)**, 20x] exhibited negative reactions.

Macroscopic examination revealed excision material measuring 3.5 × 3 × 2 cm with a smooth surface and nodular appearance. Microscopic analysis depicted a gray-white swirling pattern. The microscopic examination further revealed normocellular spindle cells displaying eosinophilic cytoplasm, cigar-shaped nuclei, and inconspicuous nucleoli. No atypia or necrosis was evident (Figures 2a, b). Very rare mitotic figures (<2/10 high power fields) were observed. Immunohistochemical assessment exhibited positive reactions with Desmin (Figure 2c) and H-Caldesmon (Figure 2d), while S-100 (Figure 2e) and CD34 (Figure 2f) showed negative reactions. Based on the histopathological and immunohistochemical findings, a diagnosis of leiomyoma was established.

### 2.3 Case 2. Vaginal leiomyoma mimicking cystocele

**Figure 3:**
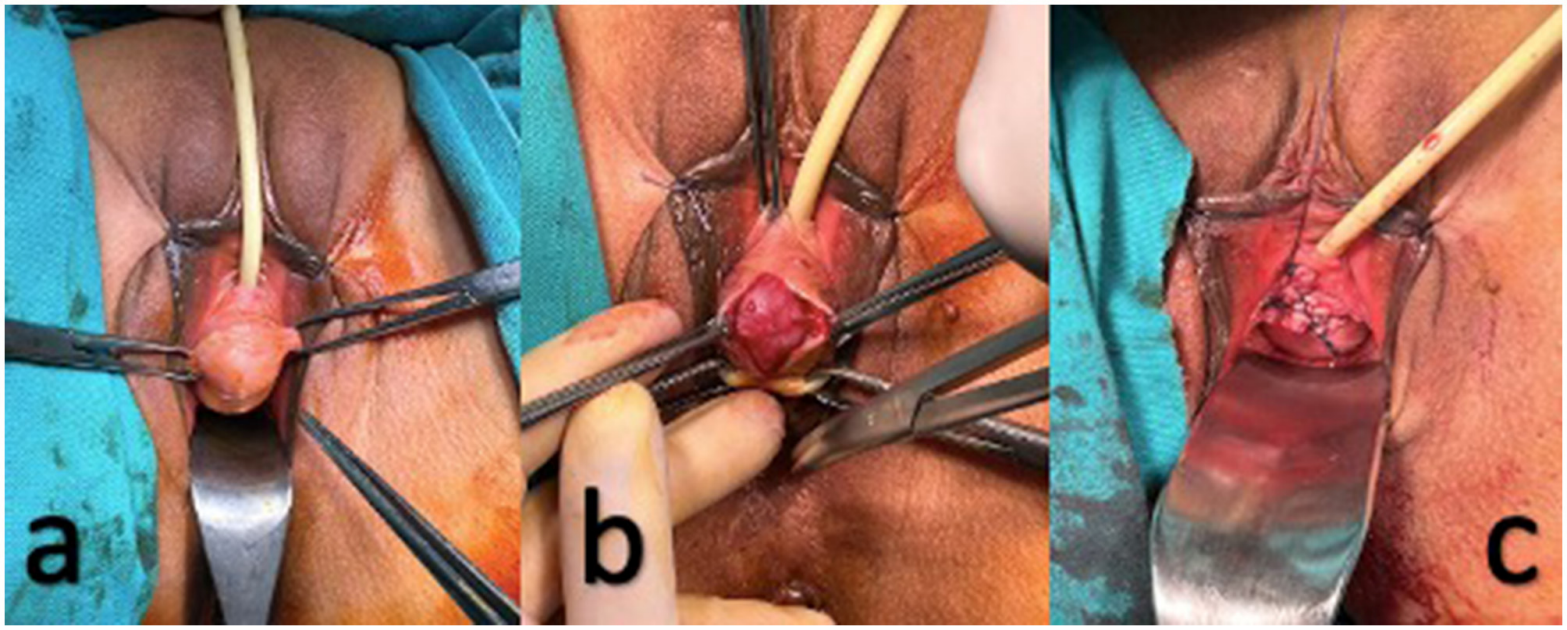
**(a)** Clinical presentation of the mass (leiomyoma) before surgery; **(b)** A mobile mass (leiomyoma) suggestive of a cystocele. **(c)** The mass (leiomyoma) was excised intact.

A 36-year-old woman, gravida 3, parity 3, was admitted to the urogynecology clinic due to the recent onset of a palpable mass on the anterior vaginal wall, accompanied by complaints of dyspareunia, dysuria and urgency. No family history of cancer, weight loss, fever, or discharge was present. She had not undergone any past interventions or surgeries. She had had three vaginal births. During the gynecology examination, a mobile mass measuring 5 × 3 × 2 cm, suggestive of a cystocele, was detected on the anterior wall of the vagina, ~1 cm away from the urethral meatus (Figure 3a). The descent of the palpable mass increased with the Valsalva maneuver. Under spinal anesthesia in the lithotomy position, the mass was completely excised via a vaginal approach from the anterior vaginal wall. The mass was excised intact and sent for histopathological analysis (Figures 3b, c). Total operation time was 30 min. There were noxperioperative or postoperativeicomplications noted. On the first day following surgery, the patient was released and experienced an uneventful recovery, resuming her daily activities within the subsequent day. She recovered well after the operation, with complete resolution of the symptoms present at the time of admission. In the 6-month follow-up time frame, no recurrence was seen.

### 2.4 Histopathological examination for Case 2

Macroscopic examination unveiled a nodular excision material measuring 5 × 3 × 2 cm with a smooth surface texture. The sections exhibited a distinctive gray-white swirling appearance, with certain regions displaying edematous and hemorrhagic characteristics. Microscopic analysis revealed normocellular spindle cells characterized by eosinophilic cytoplasm, cigar-shaped nuclei, and small nucleoli, predominantly observed throughout the specimen. Additionally, areas exhibiting pronounced edema and a heightened presence of vascular structures were identified. Notably, no atypia or necrosis were evident (Figures 2a, b). Very rare mitosis was seen (<2/10 high power field). Despite the absence of epithelial components in the histopathological examination, the incomplete architectural features, edematous stroma, and heightened vascularity observed in certain regions prompted consideration of superficial myofibroblastoma, cellular angiofibroma, angiomyofibroblastoma, and solitary fibrous tumor within the realm of the differential diagnosis. We performed an immunohistochemical panel for these differential diagnoses. We utilized Desmin, H-caldesmon, CD-34, S100, MUC4, and STAT6 in the analysis of the case. We found a diffuse strong positive reaction with Desmin (Figure 2c) and H-Caldesmon (Figure 2d) and a negative reaction with CD-34 (Figure 2f), S100 (Figure 2e), MUC4 and STAT6. Based on comprehensive evaluation incorporating both immunohistochemical and histopathological assessments, the conclusive diagnosis of leiomyoma was rendered.

## 3 Discussion

Vulvar leiomyomas are uncommon, benign, monoclonal growths that commonly occur in the fourth and fifth decades of life (7). These are typically asymptomatic benign mesenchymal tumors, rarely causing swelling and local discomfort. The majority of vulvar leiomyomas are clinically misdiagnosed as Bartholin cysts or abscesses at first (8). Unlike solid tumors, which usually present as painless masses that progressively enlarge, Bartholin's abscess appears as a painful lump that may fluctuate over time, accompanied by local inflammation and fever. On the other hand, the less painful Bartholin's cyst, is commonly linked to a background of recurring Bartholin's abscesses and might produce local discomfort. Histological evaluation is indispensable for diagnosis, particularly when clinical features hint at malignancy, such as accelerated growth (8–13).

Vaginal leiomyomas are frequently observed in women aged between 35 and 50 (14). Typically, they manifest as a solitary, well-defined mass originating from the medial anterior wall, as demonstrated in our case, and less commonly from the posterior and lateral walls (15). While often asymptomatic, they may present with chronic pelvic pain, lumbar discomfort, vaginal bleeding, dyspareunia, urinary symptoms such as pollakiuria and dysuria, or other indications of urinary obstruction, depending on their location of origin (16). Because the tumor can be mistaken for Skene's ductiabscess, urethrocele, cystocele, urethralidiverticulum, Gartner's ductxcysts, vaginalxcysts, or a malignant vaginalxtumor, a clinicalxdiagnosis of vaginal leiomyoma necessitates a high degree of skepticism (17, 18). Most of such tumors typically range in diameter from 3 to 4 cm. They are commonly solitary, benign, and characterized by slow growth. However, instances of sarcomatous transformation have been documented (19).

Various reports emphasize the importance of imaging modalities for evaluating vaginal or vulvar masses, as they can confirm the presence, location, and size of the tumor, as well as aid in its characterization. Due to its accessibility, cost-effectiveness, and non-invasive nature, the most commonly employed diagnostic technique is ultrasonography. Pelvic magnetic resonance imaging and pelvic computeditomography are employed less frequently and typically reserved for challenging occasions or in case of the presence of suspicion for malignancy or local spread. In our cases, we did not utilize any of the aforementioned imaging technologies. Given that the tumor was solitary, well-circumscribed, and lacked suspicious features upon clinical evaluation, a benign neoplasm was strongly suspected, rendering imaging investigations unnecessary. On the other hand, the role of various imaging methods in both positive and differential diagnosis of vaginal or vulvar tumors remains unclear. There is currently no consensus regarding the preferred method or defined criteria for a positive diagnosis (4, 8, 20).

Opinions regarding the risk of recurrence of vulvar or vaginal leiomyomas are contentious. Researchers advocate for long-term close monitoring due to the heightened risk of recurrence. The prevailing approach in the literature suggests that complete enucleation or excision of the tumor, along with the surrounding normal tissue, can mitigate the recurrence rate and improve the 5-year survival rate. However, owing to the limited number of instances and the scarcity of follow-upidata available, it is unknown how vulvar or vaginal leiomyomas will behave clinically over the long run (4, 20, 21).

Spindle, epithelioid, and myxoid or myxohyaline are the three primary histological patterns of vulvar or vaginal leiomyomas that have been found, while mixtures of these may also exist (4). Management is similar for all histological types. The current spindle pattern in our instances is a rather typical kind of leiomyoma, which is defined by a lot of eosinophilic cytoplasm and fascicular proliferation of spindle-shaped cells with elongated nuclei (4, 21). The histological differentiation between benign and malignant forms, such as leiomyoma, atypical leiomyoma, and leiomyosarcoma, primarily relies on a set of criteria described in the literature (4). Smooth muscle actin, desmin, and caldesmon are positive immunohistochemical markers of smooth muscle cells seen in both leiomyomas and leiomyosarcomas. But leiomyosarcomas also show immunopositivity for cytokeratin and S-100. Histological examination and immunohistochemistry can be used to distinguish leiomyoma from other mesenchymal tumors, such as aggressive angiomyxoma and cellular angiofibroma. In particular, the absence of extensive staining for smooth muscle markers, notably h-caldesmon can be used to make this distinction (22, 23). The role of estrogen, progesterone, and androgen receptors in the formation of these cancers is not well understood, but some of these tumors may express these receptors (24, 25).

In conclusion, it should be kept in mind that extrauterine leiomyomas or their malignant transformations may be present in the differential diagnosis of only conventional diagnoses in our clinical practice, such as cystocele and Bartholin cyst. Although we did not use preoperative imaging methods in our study, they can be used both in differential diagnosis and in cases of suspicion of malignancy. The primary treatment approach includes surgical excision followed by histopathological evaluation. We did not detect any recurrence in the short term in our study. We will continue to follow-up patients in the long term. There are currently no established guidelines determining their management. Additional data are needed to improve understanding of prognostic considerations, best practices for care, and diagnostic standards. In general, since clinical experience regarding the diagnosis and treatment of vulva or vaginal leiomyomas is limited, it is best to carry out diagnosis, treatment and follow-up in experienced multidisciplinary centers. We believe that multicenter studies conducted with a standardized patient management method will contribute more to the literature.

## Data availability statement

The datasets presented in this study can be found in online repositories. The names of the repository/repositories and accession number(s) can be found in the article/supplementary material.

## Ethics statement

Ethical review and approval was not required for the study on human participants in accordance with the local legislation and institutional requirements. Written informed consent was obtained from the participant/patient(s) for the publication of this case report.

## Author contributions

OY: Formal analysis, Methodology, Project administration, Resources, Validation, Visualization, Writing – original draft, Writing – review & editing. AK: Data curation, Writing – original draft. ZB: Data curation, Supervision, Writing – original draft. NA: Data curation, Writing – original draft. KM: Methodology, Writing – original draft. AA: Writing – original draft.

## References

[1] He J Liu W Wu X Li D Liu Y. A case of misdiagnosed leiomyoma of the vulva: a case report. Medicine. (2023) 102:e32868. 10.1097/MD.0000000000032868 36820583 PMC9907916

[2] Chang CH Li PC Hsu YH Ding DC. Vulvar myoma: a case report and review of literature. Taiwan J Obstet Gynecol. (2021) 60:924–6. 10.1016/j.tjog.2021.07.026 34507676

[3] Zhao T Liu X Lu Y. Myxoid epithelial leiomyoma of the vulva: a case report and literature review. Case Rep Obstet Gynecol. (2015) 2015:894830. 10.1155/2015/894830 26185695 PMC4491551

[4] Sun C Zou J Wang Q Wang Q Han L Batchu N. Review of the pathophysiology, diagnosis, and therapy of vulvar leiomyoma, a rare gynecological tumor. J Int Med Res. (2018) 46:663–74. 10.1177/0300060517721796 28875758 PMC5971502

[5] Benjelloun AT Ziad I Elkaroini D Boufettal H Mahdaoui S Samouh N. Vaginal leiomyoma mimicking a cystocele (report case). Int J Surg Case Rep. (2022) 93:106955. 10.1016/j.ijscr.2022.106955 35364392 PMC8968001

[6] Costa C Barba M Cola A Frigerio M. Transvaginal excision of vaginal paraurethral leiomyoma: a video case report. Eur J Obstet Gynecol Reprod Biol. (2023) 290:11–3. 10.1016/j.ejogrb.2023.09.008 37708657

[7] Francis SA Wilcox FL Sissons M. Bartholin's gland leiomyoma: a diagnostic and management dilemma. J Obstet Gynaecol Res. (2012) 38:941–3. 10.1111/j.1447-0756.2011.01787.x 22486791

[8] Vinturache AE Ismail L Damato S Soleymani Majd H. Challenges in management of Bartholin gland leiomyoma: a case report. Clin Case Reports. (2023) 11:1–7. 10.1002/ccr3.6449 36726693 PMC9883844

[9] Witherspoon C Garcia B Behbehani S Nahas S Stuparich MA. Vulvar leiomyoma presenting as a painless vulvar mass. J Minim Invasive Gynecol. (2022) 29:187–9. 10.1016/j.jmig.2021.10.014 34748966

[10] Heller DS. Benign tumors and tumor-like lesions of the vulva. Clin Obstet Gynecol. (2015) 58:526–35. 10.1097/GRF.0000000000000133 26125957

[11] Dole DM Nypaver C. Management of bartholin duct cysts and gland abscesses. J Midwifery Womens Health. (2019) 64:337–43. 10.1111/jmwh.12937 30734519

[12] Omole F Kelsey RC Phillips K Cunningham K. Bartholin duct cyst and gland abscess: office management. Am Fam Physician. (2019) 99:760–6. 31194482

[13] Illingworth B Stocking K Showell M Kirk E Duffy J. Evaluation of treatments for Bartholin's cyst or abscess: a systematic review. BJOG. (2020) 127:671–8. 10.1111/1471-0528.16079 31876985

[14] Chakrabarti I De A Pati S. Vaginal leiomyoma. J Midlife Health. (2011) 2:42–3. 10.4103/0976-7800.83274 21897740 PMC3156502

[15] Egbe TO Kobenge FM Metogo JAM Wankie EM Tolefac PN Belley-Priso E. Vaginal leiomyoma: medical imaging and diagnosis in a resource low tertiary hospital: case report. BMC Womens Health. (2020) 20:12. 10.1186/s12905-020-0883-2 31964370 PMC6975035

[16] Asnani M Srivastava K Gupta HP Kunwar S Srivastava AN. A rare case of giant vaginal fibromyoma. Intractable Rare Dis Res. (2016) 5:44–6. 10.5582/irdr.2015.01037 26989649 PMC4761584

[17] Sim CH Lee JH Kwak JS Song SH. Necrotizing ruptured vaginal leiomyoma mimicking a malignant neoplasm. Obstet Gynecol Sci. (2014) 57:560–3. 10.5468/ogs.2014.57.6.560 25469351 PMC4245356

[18] Wu Y Wang W Sheng X Kong L Qi J. A misdiagnosed vaginal leiomyoma: case report. Urol Case Rep. (2015) 3:82–3. 10.1016/j.eucr.2015.02.004 26793510 PMC4714314

[19] Benti TM Mercy N Eshetu AB Iwuh IE Kassa MW. Recurrent vaginal epithelioid leiomyosarcoma; a case report from Botswana and review of the literature. Afr J Reprod Health. (2021) 25:161–8. 34077121 10.29063/ajrh2021/v25i1.17

[20] Kim HR Yi BH Lee HK Hong HS Lee MH Lee HH. Vulval epithelioid leiomyoma in a pregnant woman. J Obstet Gynaecol J Inst Obstet Gynaecol. (2013) 33:210–1. 10.3109/01443615.2012.737051 23445157

[21] Tavares KAS Moscovitz T Tcherniakovsky M Pompei LM Fernandes CE. Differential diagnosis between Bartholin cyst and vulvar leiomyoma: case report. Rev Bras Ginecol Obstet. (2017) 39:433–5. 10.1055/s-0037-1604178 28783860 PMC10316947

[22] Xia C Braunstein Z Toomey AC Zhong J Rao X. S100 proteins as an important regulator of macrophage inflammation. Front Immunol. (2017) 8:1908. 10.3389/fimmu.2017.01908 29379499 PMC5770888

[23] Woida FM Ribeiro-Silva A. Adenoid cystic carcinoma of the Bartholin gland: an overview. Arch Pathol Lab Med. (2007) 131:796–8. 10.5858/2007-131-796-ACCOTB 17488169

[24] McKenzie M Pintilie H Wilkinson N Lane G Orton J El-Ghobashy A. A rare case of vulval leiomyosarcoma: management and an updated review of the literature. J Obstet Gynaecol. (2011) 31:675–6. 10.3109/01443615.2011.595519 21973156

[25] Chokoeva AA Tchernev G Cardoso JC Patterson JW Dechev I Valkanov S. Vulvar sarcomas: short guideline for histopathological recognition and clinical management. Part 2. Int J Immunopathol Pharmacol. (2015) 28:178–86. 10.1177/0394632015575977 25816393

